# Microbioma, salud y enfermedad: probióticos, prebióticos y simbióticos

**Published:** 2019-12-30

**Authors:** Arley Gómez-López

**Affiliations:** 1 Director, División de Investigaciones, Fundación Universitaria de Ciencias de la Salud, Bogotá, D.C., Colombia División de Investigaciones Fundación Universitaria de Ciencias de la Salud BogotáD.C Colombia

El hallazgo de la vida microscópica que se esconde en una gota de agua, realizado por el inventor del microscopio Antonie van Leeuwenhoek (1632-1723) en 1654, fue la puerta de entrada al estudio de la microbiología. Haciendo un paralelo, hoy podemos considerar que el avance en los métodos de secuenciación de alto rendimiento ha abierto el camino para la decodificación de genomas bacterianos de diferentes partes del cuerpo humano, base fundamental para el análisis del microbioma humano. Asimismo, el uso de nuevas tecnologías, especialmente las relacionadas con las ciencias -ómicas (genómica, transcriptómica, proteómica, metabolómica, etc.), ha permitido desarrollar proyectos como el iniciado en 2008 por investigadores de los *National Institutes of Health* (NIH) de los Estados Unidos, orientados a la comprensión de la correlación entre los cambios en el microbioma, la salud y la enfermedad en los seres humanos.

A partir del conocimiento derivado de estas investigaciones, se han desarrollado nuevas herramientas terapéuticas, tales como probióticos, prebióticos y simbióticos. Dada la importancia creciente que este tipo de productos tiene en la práctica clínica, es necesario precisar estos conceptos. La Organización Mundial de la Salud (OMS) define los probióticos como aquellos productos que contienen microrganismos vivos, los cuales, cuando se administran en la cantidad adecuada, tienen un efecto benéfico en la salud del huésped ^1^. Los prebióticos son sustancias derivadas de alimentos que no pueden ser digeridos, cuyo efecto beneficioso en el huésped está dado por su contribución al crecimiento, la actividad o ambos, de un tipo de bacterias. Los productos que contienen prebióticos y probióticos son denominados simbióticos.

## Microbioma y salud

En diversos estudios se ha demostrado que los fetos permanecen en un ambiente estéril, siempre y cuando la membrana amniótica permanezca intacta. Sin embargo, según varios estudios recientes, se han aislado bacterias simbióticas provenientes del meconio de fetos normales. Asimismo, durante el nacimiento y según la vía del parto (vaginal o cesárea), los recién nacidos adquieren bacterias por exposición a la flora vaginal y fecal materna o por microorganismos presentes en el ambiente. De hecho, se ha demostrado que los niños nacidos por cesárea tienen mayor proporción de especies y cepas, como *Bacteroides* spp., *Escherichia* spp., *Shigell*a spp. y *Clostridium difficile*^2^.

El microbioma del adulto depende de múltiples factores, como el sexo, el índice de masa corporal, los hábitos nutricionales y la actividad física, entre otros. Actualmente, el mayor número de estudios se ha centrado en la microbiota intestinal y ha evidenciado que existen más de 200 especies y subespecies de bacterias, lo cual representa entre 0,5 y 2 kg del peso corporal total de un individuo sano; no obstante, en su mayoría, corresponden a bacterias que no se pueden cultivar, lo cual dificulta su identificación por los métodos convencionales ^2^^-^^4^. La microbiota normal se ha relacionado con múltiples funciones: endocrinas, señalización neurológica, modificación de la densidad mineral ósea, maduración del sistema inmunológico, inhibición de patógenos, síntesis de vitaminas (K, B12 y folato), metabolismo de sales biliares y modulación de algunos fármacos ^5^^,^^6^.

## Microbioma y enfermedad

La correlación entre el microbioma y distintas enfermedades en el humano, se deriva de la pérdida de las funciones benéficas o de su alteración por la invasión de microrganismos patógenos. Las enfermedades más relacionadas con alteraciones de esta naturaleza incluyen: las enfermedades cardiovasculares, las autoinmunitarias e inflamatorias crónicas, la diabetes mellitus, la gastroenteritis, el síndrome de colon irritable, la artritis reumatoidea, las infecciones y el cáncer, y. también, modificaciones en el metabolismo de los fármacos.

Los principales factores asociados con riesgo cardiovascular son dislipidemia, diabetes, hipertensión arterial, obesidad y generación de trimetilamina, ácidos biliares secundarios y ácidos grasos de cadena ^7^. La trimetilamina es metabolizada por bacterias en el intestino y hace tránsito al hígado por la circulación portal, donde las monooxigenasas portadoras de flavina sintetizan el N-óxido de trimetilamina. Este compuesto se ha asociado con el desarrollo de arteriosclerosis, trombosis, falla cardiaca, fibrosis y alteraciones en la función de los macrófagos. Por otra parte, la regulación de la flora intestinal ha demostrado ser de gran beneficio en las enfermedades cardiovasculares ^8^.

En cuanto a la correlación del microbioma con el sistema inmunológico, en diversos estudios se ha planteado la relación con la autoinmunidad, las alergias y las enfermedades inflamatorias, y el tipo de gérmenes en la cavidad oral, las vías aéreas y las vías digestivas. En el caso de la artritis reumatoidea, se ha encontrado una disminución de la familia de *Bifidobacterium* y *Bacteroides*^8^. La composición de la microbiota intestinal y el estado funcional del sistema inmunológico pueden determinar cuáles pacientes padecerán la enfermedad; de hecho, algunas de las medicaciones usadas para el tratamiento de la artritis reumatoidea son antibióticos (cloroquina, sulfasalazina, minocliclina y roxitromicina) ^9^.

Se ha postulado el rol del microbioma en el desarrollo del asma y sus distintas presentaciones ^10^^,^^11^. Algunos de los mecanismos propuestos incluyen:

1. La exposición en las etapas tempranas de la vida a ambientes con gran contenido de bacterias en individuos propensos a desarrollar asma en la infancia.
2. El efecto modulador de la microbiota en el desarrollo del sistema inmunológico.
3. El efecto de las infecciones virales agudas y los patrones de colonización bacteriana de las vías respiratorias en los niños con asma.
4. La colonización bronquial por genotipos de bacterias específicos, según el fenotipo del asma.

Con respecto a las enfermedades inflamatorias, el síndrome del intestino irritable es uno de los más ampliamente estudiados y, cuando se demuestran infecciones gastrointestinales previas, se denomina síndrome de intestino irritable posinfeccioso. En este último, pueden observarse sobrepoblación bacteriana, y alteraciones cuantitativas y cualitativas de la microbiota intestinal y fecal ^12^. En el intestino delgado, se aumenta la permeabilidad intestinal y se estimulan mecanismos inmunológicos en la submucosa que derivan en una inflamación de bajo grado; incluso, esto se ha usado como justificación para usar antibióticos en su tratamiento ^12^^,^^13^.

En múltiples estudios se ha demostrado el carácter carcinogénico de microrganismos, como *Helicobacter pylori* y el virus del papiloma humano, en el cáncer gástrico y en el de cuello uterino, respectivamente. Sin embargo, recientemente se ha demostrado un aumento de la prevalencia de neoplasias con perfiles específicos de microbiota. Los microbiomas más frecuentemente asociados con neoplasias incluyen: *Fusobacterium nucleatum* en el cáncer de colon; Proteobacteria, Bacteroidetes y Firmicutes en el cáncer de páncreas; *Fusobacterium* y *Helicobacter bilis* en el cáncer de la vía biliar; *Streptococcus* spp. Y *Prevotella spp*. en EL cáncer de esófago, y *Cutibacterium acnes*, *Mycoplasma hominis*, *Enterobacteriaciae*, *Escherichia coli*, *Acinetobacter* spp. Y *Pseudomonas* spp. en el cáncer de próstata ^14^^-^^19^.

Otra de las propiedades de la microbiota es la capacidad de modificar el entorno donde se encuentra, gracias a su amplio potencial para modificar las reacciones de los fármacos, los metabolitos de los fármacos y otros xenobióticos; tal es el caso de 5-fluoruracilo a 5-fluoruridina que, mediante procesos enzimáticos, como la acetilación, alteran la estructura molecular de los fármacos y su acción.

## Probióticos, prebióticos y simbióticos

La traducción de los conocimientos generados a partir de los estudios del microbioma y su compleja interacción con el huésped, conjugada con la influencia de factores genéticos y epigenéticos, como un recurso terapéutico representado en el uso de probióticos, prebióticos y simbióticos, constituye un gran reto. A la fecha, existen múltiples estudios sobre la asociación de diversas especies y sus efectos benéficos en la salud.

El uso clínico más conocido de los probióticos es para prevenir complicaciones generadas por los antibióticos en los pacientes hospitalizados, en los cuales se ha logrado obtener una reducción hasta del 50 % de la infección por *C. difficile*^20^. Los probióticos compuestos por diversas especies a una concentración de 1011 o más unidades formadoras de colonias, utilizados por más de ocho semanas, han demostrado disminuir la presión arterial sistémica ^21^.

Uno de los productos secretados por *Streptococcus mutans* es la mutanobactina, la cual evita el crecimiento de *Candida albicans*^22^.

A pesar del número creciente de estudios en los que se ha demostrado el efecto benéfico y seguro de los probióticos, es fundamental destacar que pueden tener efectos adversos graves, e incluso letales, en algunas poblaciones vulnerables, como las mujeres embarazadas, los pacientes inmunosuprimidos, con trasplantes o con alteraciones vasculares ^23^. 

Los prebióticos y los simbióticos pueden ser una alternativa para los probióticos o parte de un complemento; de hecho, generalmente, aumentan la supervivencia en las vías digestivas de los microorganismos contenidos en los probióticos ^24^^,^^25^.

Si bien es evidente el avance logrado en el estudio de estos productos, no existe aún claridad acerca de las combinaciones ideales de microorganismos, la dosificación, la caracterización funcional y estructural de los productos secretados, los mecanismos comunes de acción para obtener su efecto benéfico, etcétera.

Dado el creciente auge de estos productos en el mercado y su potencial industrial y comercial, es un imperativo ético, científico y tecnológico, estudiar su eficacia, bioequivalencia, seguridad y vías de administración, y, en general, garantizar su uso clínico con el mayor margen de seguridad para los pacientes. Asimismo, es necesario establecer los estándares y controles de los procesos de producción que permitan garantizar la alta calidad del producto.

En cuanto al marco normativo, a excepción de los Estados Unidos, donde la *Food and Drug Administration* (FDA) considera que los estudios de los probióticos y prebióticos deben seguir el protocolo de un medicamento, en otros países se han utilizado protocolos de análisis menos rigurosos e, igualmente, metaanálisis y revisiones bibliográficas sistemáticas, como formas complementarias para evaluar su efecto benéfico ^26^^,^^27^.

En Colombia, el Ministerio de Salud y Protección Social y el Instituto Nacional de Vigilancia de Medicamentos y Alimentos (Invima) -instituciones encargadas de establecer el marco regulador de estos productos- cuentan con una normativa para la evaluación de los medicamentos con probióticos, prebióticos o simbióticos y, también, sobre los requisitos para su producción y almacenamiento. Gracias a ello, desde hace más de 10 años se han aprobado diversos productos considerados como probióticos o prebióticos, y suplementos dietarios con probióticos, simbióticos o ambos. 
